# Supplementary material for: Modeling glioblastoma heterogeneity as a dynamic network of cell states
Source: Mol Syst Biol. 2021 Sep 16;17(9):e10105. doi: 10.15252/msb.202010105 (PMC8444284; doi:10.15252/msb.202010105)
Supplement: Supplementary file 6 — Source Data for Figure 5 [file MSB-17-e10105-s004.zip › Figure5A_sourcedata/GSEA_3017/nosigdb_stateB.GseaPreranked.1622795021725/IVY_CT.html]

Details for gene set IVY\_CT[GSEA]

|  || Dataset | state43017 |
| Phenotype | NoPhenotypeAvailable |
| Upregulated in class | na\_pos |
| GeneSet | IVY\_CT |
| Enrichment Score (ES) | 0.6450234 |
| Normalized Enrichment Score (NES) | 3.6017697 |
| Nominal p-value | 0.0 |
| FDR q-value | 0.0 |
| FWER p-Value | 0.0 |
Table: GSEA Results Summary

  

Fig 1: Enrichment plot: IVY\_CT      
 Profile of the Running ES Score & Positions of GeneSet Members on the Rank Ordered List

  

| PROBE | GENE SYMBOL | GENE\_TITLE | RANK IN GENE LIST | RANK METRIC SCORE | RUNNING ES | CORE ENRICHMENT || 1 | HIST1H1B |  |  | 1 | 1.272 | 0.0275 | Yes |
| 2 | HIST1H1D |  |  | 2 | 1.180 | 0.0544 | Yes |
| 3 | TOP2A |  |  | 3 | 1.079 | 0.0789 | Yes |
| 4 | HIST1H2AH |  |  | 6 | 0.883 | 0.0961 | Yes |
| 5 | RRM2 |  |  | 7 | 0.875 | 0.1160 | Yes |
| 6 | UBE2C |  |  | 11 | 0.802 | 0.1298 | Yes |
| 7 | TPX2 |  |  | 12 | 0.801 | 0.1481 | Yes |
| 8 | NUSAP1 |  |  | 13 | 0.801 | 0.1663 | Yes |
| 9 | HMGB2 |  |  | 14 | 0.786 | 0.1842 | Yes |
| 10 | FBXO5 |  |  | 15 | 0.785 | 0.2021 | Yes |
| 11 | NCAPG |  |  | 16 | 0.778 | 0.2198 | Yes |
| 12 | PRC1 |  |  | 18 | 0.756 | 0.2355 | Yes |
| 13 | CDCA2 |  |  | 19 | 0.751 | 0.2526 | Yes |
| 14 | ESCO2 |  |  | 20 | 0.742 | 0.2695 | Yes |
| 15 | TACC3 |  |  | 21 | 0.741 | 0.2864 | Yes |
| 16 | GTSE1 |  |  | 22 | 0.732 | 0.3031 | Yes |
| 17 | LRRN3 |  |  | 25 | 0.704 | 0.3161 | Yes |
| 18 | ASPM |  |  | 26 | 0.695 | 0.3320 | Yes |
| 19 | NDC80 |  |  | 27 | 0.695 | 0.3478 | Yes |
| 20 | KIF15 |  |  | 29 | 0.684 | 0.3619 | Yes |
| 21 | CENPF |  |  | 34 | 0.670 | 0.3713 | Yes |
| 22 | KIF4A |  |  | 37 | 0.662 | 0.3834 | Yes |
| 23 | RAD51AP1 |  |  | 40 | 0.657 | 0.3954 | Yes |
| 24 | SMC4 |  |  | 41 | 0.655 | 0.4103 | Yes |
| 25 | MELK |  |  | 42 | 0.655 | 0.4252 | Yes |
| 26 | CENPE |  |  | 43 | 0.654 | 0.4401 | Yes |
| 27 | NUF2 |  |  | 44 | 0.651 | 0.4549 | Yes |
| 28 | DLGAP5 |  |  | 46 | 0.647 | 0.4682 | Yes |
| 29 | DHFR |  |  | 49 | 0.638 | 0.4798 | Yes |
| 30 | AURKA |  |  | 55 | 0.617 | 0.4865 | Yes |
| 31 | FANCI |  |  | 56 | 0.615 | 0.5005 | Yes |
| 32 | LPL |  |  | 57 | 0.615 | 0.5145 | Yes |
| 33 | MYBL2 |  |  | 58 | 0.612 | 0.5284 | Yes |
| 34 | CKAP2L |  |  | 59 | 0.611 | 0.5423 | Yes |
| 35 | CENPK |  |  | 62 | 0.603 | 0.5531 | Yes |
| 36 | BUB1B |  |  | 64 | 0.596 | 0.5652 | Yes |
| 37 | KIF2C |  |  | 66 | 0.591 | 0.5772 | Yes |
| 38 | CDK1 |  |  | 69 | 0.585 | 0.5876 | Yes |
| 39 | CDCA8 |  |  | 82 | 0.562 | 0.5828 | Yes |
| 40 | KIF14 |  |  | 85 | 0.557 | 0.5925 | Yes |
| 41 | CENPM |  |  | 92 | 0.544 | 0.5961 | Yes |
| 42 | PBK |  |  | 95 | 0.540 | 0.6055 | Yes |
| 43 | CDCA3 |  |  | 111 | 0.511 | 0.5951 | Yes |
| 44 | BUB1 |  |  | 113 | 0.508 | 0.6052 | Yes |
| 45 | BIRC5 |  |  | 116 | 0.504 | 0.6138 | Yes |
| 46 | CLSPN |  |  | 117 | 0.503 | 0.6252 | Yes |
| 47 | ASF1B |  |  | 120 | 0.501 | 0.6337 | Yes |
| 48 | HIST1H2AM |  |  | 121 | 0.497 | 0.6450 | Yes |
| 49 | TTK |  |  | 137 | 0.482 | 0.6340 | No |
| 50 | HIST1H2AG |  |  | 143 | 0.466 | 0.6373 | No |
| 51 | CDKN2C |  |  | 164 | 0.445 | 0.6181 | No |
| 52 | CDC20 |  |  | 184 | 0.431 | 0.6001 | No |
| 53 | CCNE2 |  |  | 215 | 0.406 | 0.5653 | No |
| 54 | EZH2 |  |  | 236 | 0.388 | 0.5448 | No |
| 55 | CCNB2 |  |  | 250 | 0.379 | 0.5344 | No |
| 56 | RAMP1 |  |  | 285 | 0.357 | 0.4927 | No |
| 57 | DTL |  |  | 305 | 0.345 | 0.4727 | No |
| 58 | FAM111B |  |  | 314 | 0.342 | 0.4687 | No |
| 59 | OIP5 |  |  | 315 | 0.341 | 0.4765 | No |
| 60 | KCNIP1 |  |  | 317 | 0.339 | 0.4827 | No |
| 61 | NES |  |  | 334 | 0.332 | 0.4668 | No |
| 62 | LMNB1 |  |  | 363 | 0.321 | 0.4331 | No |
| 63 | ID3 |  |  | 389 | 0.311 | 0.4035 | No |
| 64 | BLM |  |  | 408 | 0.305 | 0.3841 | No |
| 65 | PTTG1 |  |  | 413 | 0.302 | 0.3851 | No |
| 66 | TTYH1 |  |  | 469 | 0.287 | 0.3110 | No |
| 67 | RNASEH2A |  |  | 478 | 0.285 | 0.3057 | No |
| 68 | DNMT1 |  |  | 523 | 0.277 | 0.2475 | No |
| 69 | SAPCD2 |  |  | 578 | 0.268 | 0.1745 | No |
| 70 | UHRF1 |  |  | 652 | 0.255 | 0.0732 | No |
| 71 | METRN |  |  | 695 | -0.273 | 0.0179 | No |
| 72 | ID4 |  |  | 708 | -0.285 | 0.0068 | No |
| 73 | DSEL |  |  | 709 | -0.297 | 0.0135 | No |
| 74 | OLIG1 |  |  | 729 | -0.370 | -0.0059 | No |
| 75 | DLL3 |  |  | 736 | -0.414 | -0.0053 | No |
| 76 | DBI |  |  | 740 | -0.422 | -0.0001 | No |
| 77 | HES6 |  |  | 752 | -0.523 | -0.0043 | No |
| 78 | SOX11 |  |  | 754 | -0.576 | 0.0073 | No |
Table: GSEA details [plain text format]

  

Fig 2: IVY\_CT: Random ES distribution      
 Gene set null distribution of ES for **IVY\_CT**
